# Melatonin-Mediated Sugar Accumulation and Growth Inhibition in Apple Plants Involves Down-Regulation of Fructokinase 2 Expression and Activity

**DOI:** 10.3389/fpls.2019.00150

**Published:** 2019-02-19

**Authors:** Jingjing Yang, Chunxia Zhang, Zhengyang Wang, Simin Sun, Ruiling Zhan, Yuyue Zhao, Baiquan Ma, Fengwang Ma, Mingjun Li

**Affiliations:** ^1^State Key Laboratory of Crop Stress Biology for Arid Areas, Shaanxi Key Laboratory of Apple, College of Horticulture, Northwest A&F University, Yangling, China; ^2^College of Forestry, Northwest A&F University, Yangling, China

**Keywords:** apple, fructokinase, growth inhibition, *MdFRK2*, melatonin, sugar

## Abstract

Melatonin has been reported to play roles in regulating carbohydrate levels and plant growth. However, little is known about the exact mechanism by which melatonin regulates sugar levels and growth in plants. In this study, it was found that high levels of melatonin inhibited the growth of wild-type (WT) apple plants and induced significant accumulations of fructose, glucose, and sucrose in apple leaves, while *MdFRK2* expression was significantly downregulated. *MdFRK2* promoter transiently expressed in tobacco leaves further supported that the expression of *MdFRK2* could be inhibited by exogenous melatonin. After applying exogenous melatonin, the suppression of *MdFRK2* expression was significantly rescued in transgenic apples overexpressing *MdFRK2* via the 35S promoter. Fructose, glucose, and sucrose concentrations increased less as compared to WT apple plants. Wild-type plants showed a stunted phenotype 21 days after melatonin treatment, while *MdFRK2*-overexpressing plants exhibited slightly inhibited growth, indicating that the downregulated *MdFRK2* expression in response to melatonin was involved in melatonin-mediated growth inhibition. Taken together, these results demonstrate the involvement of *MdFRK2* in melatonin-induced sugar accumulation and growth inhibition. Our findings shed light on the roles played by *MdFRK2* in connecting melatonin action and plant growth.

## Introduction

Melatonin (*N*-acetyl-5-methoxytryptamine), a small, highly conserved molecule involved in the process of biological evolution, is widely present in organisms including bacteria, unicellular eukaryotes, macroalgae, fungi, higher plants, invertebrates, and vertebrates (Tan et al., 2010). Although melatonin is known mainly as an animal hormone and neurotransmitter, its role in plants is currently being extensively investigated (Erland et al., 2015). Melatonin acts as a plant growth and development regulator as well as a biostimulant or perhaps even as a DAMP when some cells are leaking as a consequence of (a) biotic stresses (Hardeland et al., 2012; Versluys et al., 2017). It plays a role in the plant photoperiod response, scavenging of reactive oxygen species and protection of plants against bacterial pathogens by activating expression of defense-related genes (Park et al., 2013; Wang et al., 2013, 2015; Liang et al., 2015; Qian et al., 2015; Mandal et al., 2018). As a result, exogenous melatonin is widely used to improve resistance against biotic/abiotic stress or to regulate plant growth. Although one melatonin receptor has been identified in *Arabidopsis thaliana* (Wei et al., 2018), the understanding of the functional mechanisms of melatonin action in plants is very limited.

Exogenous melatonin significantly affects carbohydrate/sugar concentrations in maize (*Zea mays*) (Zhao et al., 2015a), apple (*Malus*) (Wang et al., 2015), *Arabidopsis* (Zhao et al., 2015b), and *Prunus avium* × *Prunus cerasus* (Sarropoulou et al., 2012). Carbohydrate fuels all the processes of plant metabolic pathways and satisfies the extra requirements for ATP, NADPH, and other metabolites. Soluble sugar molecules such as sucrose, glucose (Rolland et al., 2006) and fructose (Cho and Yoo, 2011) act as signaling molecules that regulate the expression of both various metabolism- and defense-related genes and metabolic processes that control plant growth and development (Rolland et al., 2006; Janse van Rensburg et al., 2019). The novel ‘sweet immunity’ concept predicts that sweet, endogenous saccharides such as sucrose, raffnose family oligosaccharides, fructans, and galactinol may act as signaling molecules that are activated by exposure to stress and hence initiate signal amplification and lead to more rapid and robust activation of defense, immunity and stress tolerance (Bolouri Moghaddam and Van den Ende, 2012, 2013; Versluys et al., 2017). As a signaling molecule, hexose accumulation in leaves represents the onset of senescence and inhibits growth, as this accumulation tends to repress photosynthesis (Cho and Yoo, 2011). As reported (Zhao et al., 2015a), high doses of melatonin stopped seedling growth and caused leaf senescence by inducing excessive accumulations of sucrose, hexose, and starch in the leaves of maize seedlings.

In plant cells, sugar concentrations are highly regulated by sugar metabolism, which involves the breakdown of sucrose by invertase and sucrose synthase (SUSY), the phosphorylation of the resulting hexoses and the interconversion between hexose phosphates and UDP-glucose, and the synthesis of sucrose via SPS and SPP (Pego and Smeekens, 2000; Ruan, 2014; Li et al., 2018). In maize seedlings, sucrose synthesis and hydrolysis increased in response to exogenous melatonin application, as reflected by the elevated gene expression and enzymatic activities of SPS and acid invertase (Zhao et al., 2015a). However, the expression of *FRK2*, a gene encoding fructokinase (FRK), which phosphorylates fructose to fructose-6-phosphate (F6P), was downregulated in both the source and sink parts of the maize leaves, especially under high concentrations of melatonin applications (Zhao et al., 2015a).

Fructokinase is the gateway to fructose metabolism, and *FRK2* orthologs are the major fructose-phosphorylating high-affinity enzymes in tomato (Kanayama et al., 1997, 1998), rice (*Oryza sativa*) (Jiang et al., 2003), maize (Zhang et al., 2003), and potato (*Solanum tuberosum*) (Renz and Stitt, 1993). Suppression of the *FRK2* orthologs resulted in phenotypes of plants (increased sugar concentrations and inhibited growth) that were similar to those of plants treated with high concentrations of melatonin. For example, antisense suppression of the *FRK2* ortholog (*StFRK1*) in potato resulted in increased levels of sugars (fructose, glucose, sucrose) and reduced aerial growth (Davies et al., 2005). In tomato, RNA interference (RNAi)*-LeFRK2* plants exhibited diminished stem growth and wilted leaves—phenotypes that are related to a reduction in the area of active xylem in the stem (German et al., 2003). *RNAi-FRK2* aspen plants also presented elevated concentrations of fructose, glucose, and sucrose and decreased cell wall fiber thickness due to the decreased carbon flux to cell wall polysaccharide precursors (Roach et al., 2012). Additionally, our results showed that overexpressing *MdFRK2* in apple decreased the concentrations of fructose, glucose, and sucrose while both SPS and neutral invertase expression and enzyme activity decreased (Yang et al., 2018). Overall, there seems to be an intimate link between *FRK2* expression and melatonin application. It can be speculated that *FRK2* is involved in melatonin-mediated variations in sugar metabolism and stunted growth.

To test this hypothesis, we investigated the influence of exogenous melatonin on sugar concentrations and the expression of genes related to sugar metabolism in mature leaves of apple. The *MdFRK2* promoter coupled to a β-glucuronidase (GUS) reporter gene was transiently expressed in tobacco leaves to further confirm the influence of melatonin on *MdFRK2* expression. Last, transgenic apple lines overexpressing *MdFRK2* were used to further investigate the role of *MdFRK2* in regulating sugar concentrations and plant growth under exogenous melatonin application. In this study, our results show that melatonin regulates sugar accumulation and plant growth by inhibiting *FRK2* expression, providing new insights into the roles played by melatonin in regulating sugar concentrations in plants.

## Materials and Methods

### Plant Materials and Treatments

Wild-type *Malus domestica* cv. ‘Royal Gala’ tissue-cultured plants were initially grown on an MS medium supplemented with 0.2 mg L^−1^ IAA and 0.3 mg L^−1^ 6-BA for 4 weeks. They were then transferred to a rooting medium (MS + 0.5 mg IBA and 0.5 mg IAA) for about 60 days. After rooting, the tissue-cultured plants were transplanted to small black plastic pots with good drainage (12 cm × 12 cm) that were filled with soil and sand, and were transferred to a culture room maintained at 23°C and a 14-h photoperiod supplemented with fluorescent light (60 μM m^−2^ s^−1^). One month after acclimating to controlled conditions, plants of similar growth and size were selected for exogenous melatonin treatments. The control was, supplied with thirty milliliters water every 3 days. Treatments received thirty milliliters melatonin solution at concentrations of 0, 100, 500, 1,000, and 5,000 μM as described before (Wang et al., 2013), applied to the soil in each pot every 3 days for 14 days. Afterward, plant heights were measured, and the fourth to seventh mature leaves from the base of the stem (fully mature leaves) were harvested and stored at −80°C.

We obtained *MdFRK2-*OE transgenic apple lines (L1, L4, and L9) with the cauliflower mosaic virus 35S promoter (CaMV 35S) promoter (Supplementary Figure S1). Compared to untransformed WT lines, *MdFRK2* expression increased by 9.2-, 13.0-, and 13.2-fold in lines L1, L4, and L9, respectively. To test whether *MdFRK2* is involved in melatonin-mediated variations in sugar metabolism and growth inhibition, *MdFRK2*-OE tissue-cultured plants (L1, L4, and L9) were selected to receive exogenous melatonin treatments. The growth and culture conditions of the transgenic plants are the same as those of WT described above. In a previous study, 1,000 μM of melatonin initially had an inhibitory effect on the growth of WT plants, so we selected 1,000 μM as the most appropriate concentration for the treatment of the *MdFRK2*-OE lines. Healthy and uniform plants of WT and transgenic lines (L1, L4, and L9) were selected to receive melatonin application. Regarding the WT and transgenic lines (L1, L4, and L9), control group was irrigated with standard water, and another group was irrigated with 1,000 μM of melatonin dissolved in the irrigation water. The treatment method was similar to that mentioned above. We recorded the day on which the melatonin treatment was started as the first day, and the plant height was measured every 7 days.

Tobacco plants (*Nicotiana tabacum* L., ‘Samsun NN’) were grown at 25°C, and the 16-h photoperiod was supplemented with lamps at 120 μM m^−2^ s^−1^. After 1 month, 35 plants were separated into seven groups (one group consisted of at least four plants as one biological replicate). Five groups were irrigated with different concentrations of exogenous melatonin (10, 100, 500, 1,000, or 5,000 μM) while another two groups were well-watered as controls.

### Analysis of Soluble Sugars

Soluble sugars were obtained and derivatized according to the method of Yang et al. (2018). Each sample (0.1 g) was extracted in 1.4 ml of 75% methanol, and ribitol was added as an internal standard. After fractionation of non-polar metabolites in chloroform, 5 μL of the polar phase was transferred to 2.0 ml Eppendorf vials to measure the metabolites in each sample, such as sorbitol, sucrose, glucose, and fructose. The metabolites were dried under vacuum without heating and then derivatized with methoxyamine hydrochloride and *N*-methyl-*N*-trimethylsilyl-trifluoroacetamide sequentially. Afterward, the metabolites were analyzed with a Shimadzu GCMS-2010SE device (Shimadzu Corporation, Kyoto, Japan). The metabolites were identified by comparing their fragmentation patterns with those from a mass spectral library generated on our GC/MS system and those from an annotated quadrupole GC/MS spectral library downloaded from the Golm Metabolome Database^1^. The quantifications were based on standard curves generated for each metabolite and internal standard.

**FIGURE 1:**
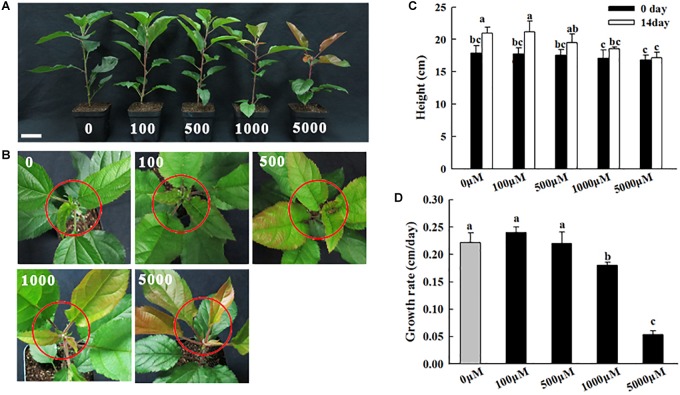
Phenotypes of apple plants under different concentrations of melatonin treatment (0, 100, 500, 1,000, and 5,000 μM). **(A)** Growth performance, **(B)** shoot tip phenotypes, **(C)** stem height, and **(D)** growth rate were recorded after 14 days of treatment. The plant height was considered the average of 6 plants per treatment. The red circles indicate the shoot tips. Values are means of four replicates SD. Different letters above the column indicate significant difference at *P* < 0.05 by Duncan’s test. Scale bars represent 5 cm.

**FIGURE 2:**
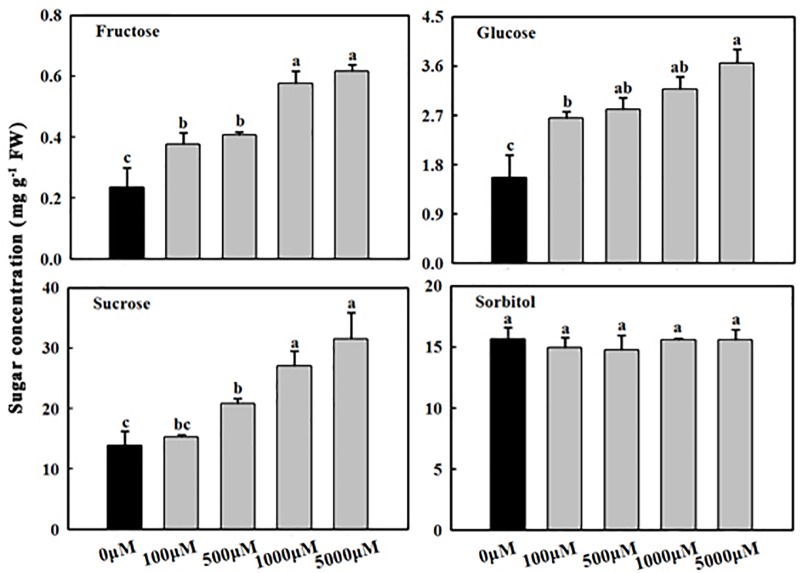
Effects of exogenous melatonin on fructose, glucose, sucrose, and sorbitol concentrations in apple leaves. Samples were obtained from fully mature leaves (the fourth to seventh leaves from the base of the stem) of WT plants after 14 days of melatonin applications (0, 100, 500, 1,000, and 5,000 μM). Values are means of four replicates ± SD. Different letters above the column indicate significant difference at *P* < 0.05 by Duncan’s test.

### RNA Extraction and qRT-PCR Assays

The total RNA was extracted from each sample using an RNAprep plant kit (Tiangen, Beijing, China), according to the manufacturer’s instructions. The RNA was then reverse-transcribed into cDNA with a PrimeScript^TM^ RT reagent kit and gDNA Eraser to avoid possible genomic DNA contamination (TaKaRa, Dalian, China). The primer sequences used for quantitative real-time polymerase chain reaction (qRT-PCR) assays are presented in Supplementary Table S1. *Mdactin* (CN938023) was chosen as an internal control for data normalization. qRT-PCR was performed on an ABI 7300 Real-Time PCR instrument (Thermo Fisher Scientific) using a SYBR Green Premix Ex Taq Kit (TaKaRa, Kyoto, Japan). The PCR conditions were as follows: predenaturing at 94°C for 5 min followed by 40 cycles of 95°C for 15 s and 58°C for 60 s. For each sample, total RNA was extracted from three biological replicates. The data were analyzed using the ddCT method.

### Assays of Enzyme Activities

The enzymes in the mature leaves of WT and transgenic lines were extracted as described by Li et al. (2012), with some modifications. Briefly, a 0.5 g sample was homogenized in 2 ml of 200 mM Hepes-KOH (pH 8.0) buffer, containing 5 mM MgCl_2_, 2 mM EDTA, 2.5 mM dithiothreitol (DTT), 2 mM benzamidine, 2 mM ε-aminocaproic acid, 0.1 mM 4-(2-aminoethyl)-benzenesulfonyl fluoride (AEBSF), 1% bovine serum albumin (BSA) (w/v), 2% glycerol (v/v), 0.05% Triton X-100 (v/v) and 2% polyvinylpyrrolidone (PVP) (w/v). The extract was then centrifuged at 16,000 *g* for 20 min at 4°C, after which 1 ml of the supernatant was immediately desalted in a Sephadex G25 PD-10 column (GE Healthcare, United Kingdom) and subsequently equilibrated with the above mentioned extraction buffer containing a concentration of 50 mM Hepes-KOH (pH 7.4) but without Triton X-100, DTT, BSA, and PVP.

We determined the activity of SPS as described by Li et al. (2016). The assay mixture (200 μl) contained 50 mM Hepes-KOH (pH 7.4), 4 mM MgCl_2_, 1 mM EDTA, 4 mM F6P, 3 mM UDP-glucose, 20 mM G6P and 100 μl of the sample. The reaction was carried out at 27°C for 30 min, then it was stopped by boiling in water for 3 min. The blank was run for each assay by adding denatured extracts. After centrifugation for 2 min at 12,000 *g*, 75 μl of the reaction mixture was used for UDP measurements in a spectrophotometric assay. The assay mixture (1.0 mL) contained 50 mM Hepes-KOH (pH 7.0), 0.8 mM phosphoenolpyruvate, 0.3 mM NADH, 5 mM MgCl_2_, 14 U of lactate dehydrogenase, and 4 U of pyruvate kinase (to start the reaction).

Neutral invertase was incubated at 37°C for 60 min in a 200 μl assay mixture that contained 100 mM phosphate-citrate buffer (pH 7.2), 100 mM sucrose, and 50 μl of the desalted extract or denatured extract (as a blank). The assay was stopped by boiling for 3 min before adding 0.75 M Tris-HCl buffer (pH 8.5). The blanks contained the same mixture, but the extract was boiled for 5 min before being mixed. The level of glucose produced from sucrose was determined by the enzyme-coupling method (Li et al., 2016).

Sorbitol dehydrogenase activity was assayed in a 1.0 ml reaction mixture that contained 1 mM NAD^+^, 300 mM sorbitol, and 0.2 ml of desalted extract in 100 mM Tris-HCl (pH 9.6), and NADH production was determined spectrophotometrically at 340 nm.

Hexokinase (HXK) and FRK activities were assayed via a continuous spectrophotometric assay as described by Li et al. (2016), with minor modifications. For HXK, the 0.5 ml assay mixture contained 50 mM Tris-HCl (pH 8.0), 2.5 mM ATP, 4 mM MgCl_2_, 1 U of G6P dehydrogenase, 0.33 mM NAD^+^, 1 mM glucose and 25 ml of desalted extract. For FRK, one unit of phosphoglucoisomerase was also added, and 0.4 mM fructose was used instead of glucose.

### Promoter Cloning and GUS Activity Analysis

Based on the genome contig sequence of MDC019777.147, we designed primers to clone the promoter of *MdFRK2* (Supplementary Table S1). As the promoter sequence, the upstream 1.8 kb region of *MdFRK2* mRNA was cloned from the genomic DNA of ‘Royal Gala’ apple. To examine the transient gene expression in tobacco, the *MdFRK2* promoter (P*MdFRK2*) was introduced into a pC0390GUS vector to generate P*MdFRK2*-GUS. The empty pC0390GUS vector served as a negative control. *Agrobacterium*-mediated transient transformation was conducted with samples of fully expanded tobacco leaves in accordance with a previously described protocol (Sparkes et al., 2006).

At 48 h after infiltration, the tissues were irrigated with 25 mL of different concentrations of exogenous melatonin (0, 10, 100, 500, 1,000, or 5,000 μM). After 12 h of irrigation, leaves were stained for 2 h using a GUS histochemical assay as previously described (Xu et al., 2011), while two leaves from each plant were harvested to analyze the GUS activity.

### Statistical Analysis

The data were expressed as the means ± standard deviations (SDs) of three replicate samples per measurement. All statistical analyses were performed using IBM SPSS Statistics 21 software (SPSS, Inc., Chicago, IL, United States), and graphs were generated with Sigma Plot 12.5. The data were analyzed using one-way ANOVA and Duncan’s multiple range test, where differences were considered significant at *P* < 0.05.

**FIGURE 3:**
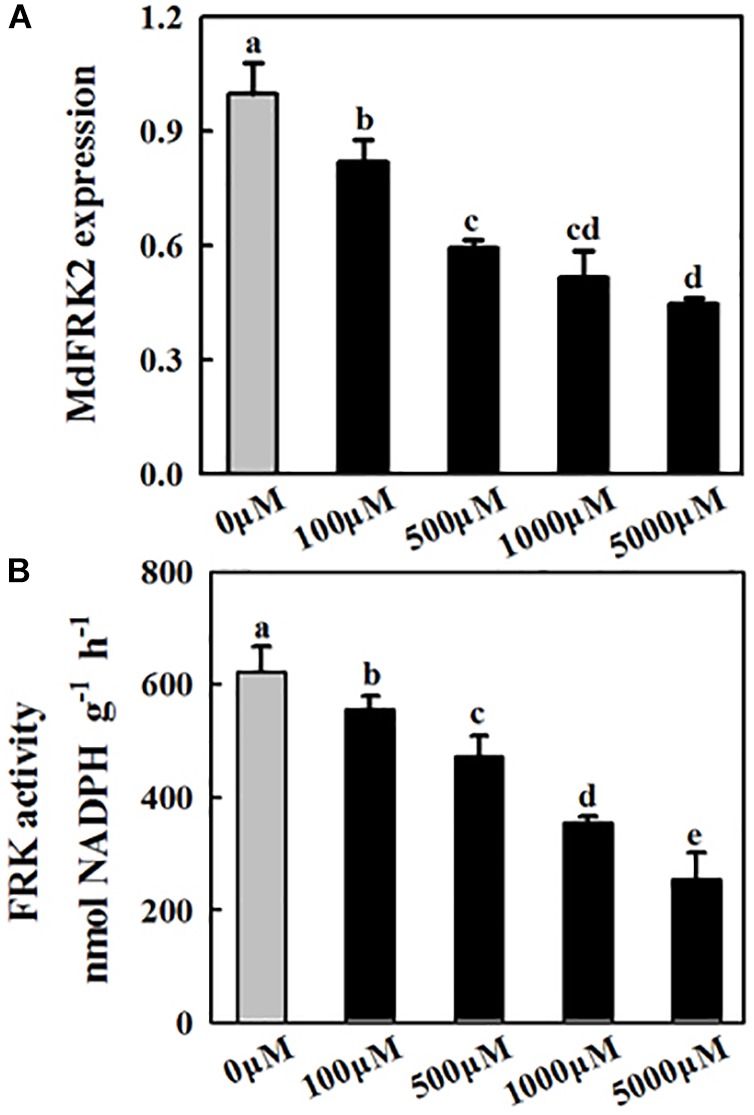
Effects of exogenous melatonin on *MdFRK2* expression **(A)** and FRK activity **(B)** in apple leaves. Samples were obtained from fully mature leaves (the fourth to seventh leaves from the base of the stem) of WT plants after 14 days of melatonin applications (0, 100, 500, 1,000, and 5,000 μM). The total RNA was isolated, and qRT-PCR was performed with gene-specific primers. For the qRT-PCR assay, the *MdActin* gene served as an internal control. Values are means of four replicates ± SD. Different letters above the column indicate significant difference at *P* < 0.05 by Duncan’s test.

**FIGURE 4:**
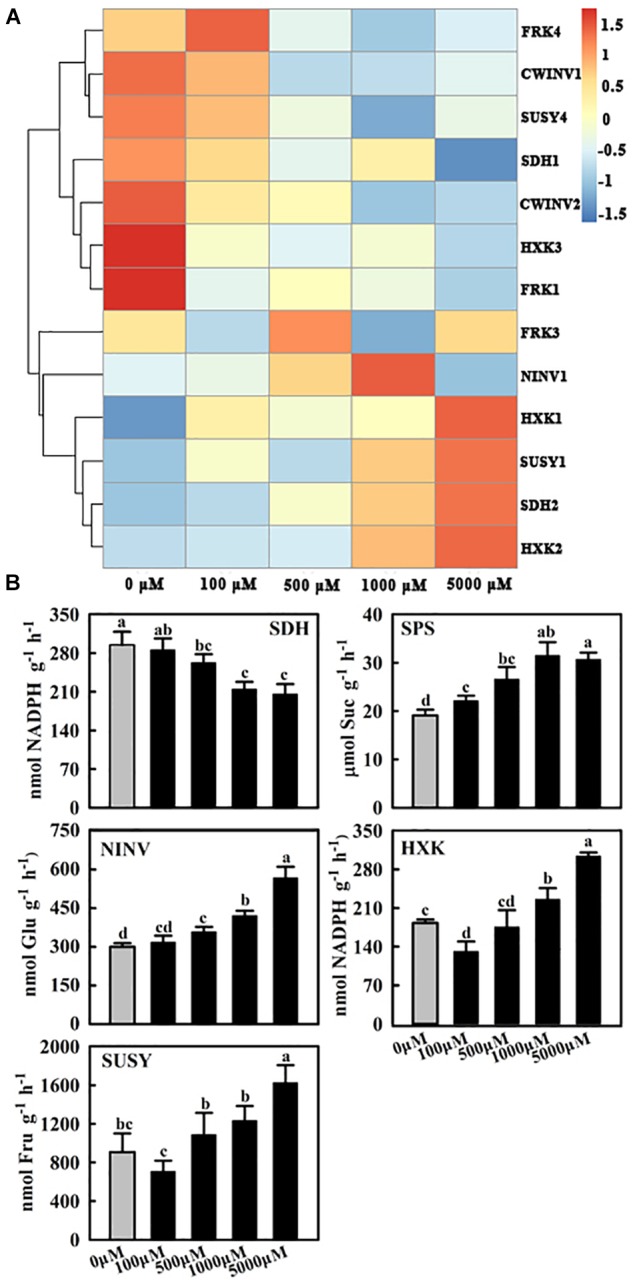
Effects of exogenous melatonin on the relative mRNA expression of genes **(A)** and activities of enzymes **(B)** involved in sugar metabolism. Samples were obtained from fully mature leaves (the fourth to seventh leaves from the base of the stem) of WT plants after 14 days of melatonin applications (0, 100, 500, 1,000, and 5,000 μM). The expression was normalized to that of *MdActin* mRNA. The data represent the means ± SD of three replicates. SPS, sucrose-phosphate synthase; NINV, neutral invertase; SUSY, sucrose synthase; SDH, sorbitol dehydrogenase; HXK, hexokinase. Values are means of four replicates ± SD. Different letters above the column indicate significant difference at *P* < 0.05 by Duncan’s test.

## Results

### Apple Plants Growth Is Affected by Melatonin

To investigate the concentration-dependent effects of melatonin on the growth of apple plants via phenotypic analysis, 2-month-old apple plants grown in soil were irrigated with different concentrations of melatonin solution (0, 100, 500, 1,000, and 5,000 μM) for 14 days (see section “Materials and Methods”). Compared with the control treatment, the low-concentration melatonin treatments (0, 100, 500 μM) caused no significant phenotypic changes; however, the high-concentration melatonin treatments (1,000 and 5,000 μM) inhibited apple plants growth after 14 days of treatment, as reflected by the shoot height and growth rate. Compared to the control, the plant growth rates in response to the 5,000 μM melatonin treatment were reduced by 50.03% (Figure 1A,C). Additionally, when treated with high concentrations of melatonin (1,000 and 5,000 μM), the young leaves and shoot tips of plants clearly appeared red in color (Figure 1B). These observations implied that high concentrations of exogenous melatonin inhibited stem growth and induced leaf anthocyanin accumulations.

### Sugar Metabolism in Mature Apple Leaves Is Affected by Melatonin

As reported previously (Wang et al., 2013; Zhao et al., 2015a), the concentrations of sucrose, glucose, and fructose in the melatonin treatment group were higher than those in the control (water) treatment group (Figure 2). The melatonin concentrations were positively correlated with the sugar concentrations (sucrose, glucose, and fructose) (Figure 2). Compared to the control, the concentrations of fructose, glucose, and sucrose in mature leaves treated with 1,000 μM of melatonin increased 2.47-, 2.02-, and 1.95-fold, respectively. However, sorbitol concentrations did not significantly differ between the treated groups and the control group.

### The Expression of Genes and Enzymes Related to Sugar Metabolism Is Affected by Melatonin

To understand how melatonin regulates carbohydrates in apple mature leaves, the expression of key genes and the activities of enzymes involved in sugar metabolism were subsequently assessed. The transcript levels of *MdFRK2* clearly decreased as the melatonin concentrations increased (Figure 3A), and FRK activity showed a similar trend (Figure 3B). NINV activity was significantly upregulated after melatonin treatment, although the expression of *MdNINV1* was inhibited by melatonin (Figure 4A,B). The activity of SPS, the major enzyme participating in the sucrose biosynthesis pathway, was also upregulated after melatonin treatment, especially in response to the 1,000 and 5,000 μM treatments (Figure 4B). SDH, the key enzyme regulating sorbitol degradation, was downregulated after melatonin application. HXK activity raised gradually with the increase of melatonin concentration (Figure 4B).

### Melatonin Inhibits the Activity of the *MdFRK2* Promoter

**FIGURE 5:**
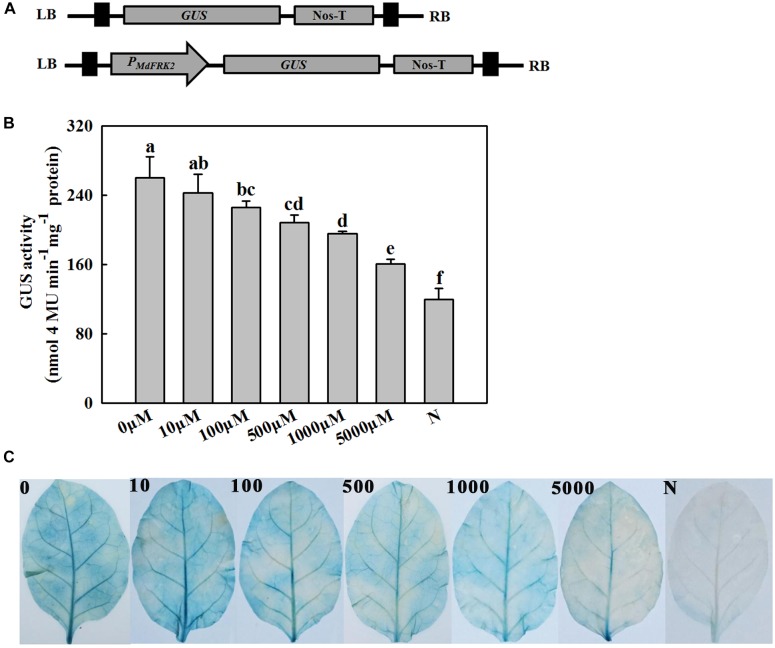
Analysis of the activity of the MdFRK2 promoter under melatonin treatment. Tobacco that transiently expressed the *MdFRK2* promoter was irrigated with different concentrations of melatonin (0, 100, 500, 1,000, and 5,000 μM). **(A)** Schematic diagram of the vector construct. The *MdFRK2* promoter (P*MdFRK2*) was cloned into pC0390GUS and fused with a GUS reporter. P0-GUS (empty pC0390GUS vector) served as a negative control. GUS, β-glucuronidase; LB, left border; NOS-T, Nos terminator; RB, right border. **(B)** Activity of the MdFRK2 promoter inhibited by melatonin. The vector P*MdFRK2*-GUS was transiently expressed in tobacco leaves by the *Agrobacterium*-mediated method, and the tobacco was then irrigated with different concentrations of melatonin (0, 10, 100, 500, 1,000, and 5,000 μM). The GUS activity in leaf extracts was measured using a microplate spectrophotometer. Values are means of three replicates ± SD. Different letters above the column indicate significant difference at *P* < 0.05 by Duncan’s test. **(C)** Leaf phenotypes in response to different concentrations of melatonin treatments.

To examine the hypothesis that the expression of *MdFRK2* is inhibited by melatonin, a β-glucuronidase (GUS) reporter gene was fused downstream from the *MdFRK2* promoter (MdFRK2P), and then the resulting MdFRK2P::GUS construct was introduced into tobacco by *Agrobacterium*-mediated transient transformation (Figure 5A). The transgenic plants were treated with different concentrations of melatonin (0, 10, 100, 500, 1,000, and 5,000 μM), after which histochemical staining was performed. As shown in Figure 5B, the transgenic plants treated with low concentrations of melatonin showed a strong blue precipitate, while high concentrations of melatonin showed a slight blue precipitate (Figure 5B). To accurately measure the level of GUS activity, quantitative GUS activity assays were performed. The highest activity occurred in the transgenic lines treated with 10 μM of melatonin (Figure 5C), but the *MdFRK2* promoter activity decreased as exogenous melatonin concentrations in the transgenic lines increased. These results suggested that the *MdFRK2* promoter activity could be inhibited by melatonin.

**FIGURE 6:**
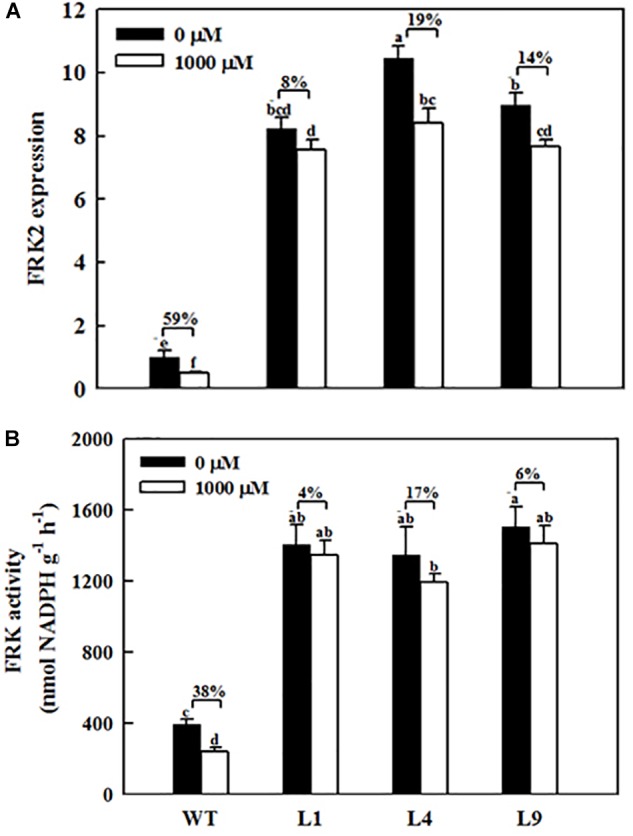
Effects of exogenous melatonin on *MdFRK2* expression **(A)** and FRK activity **(B)** in mature leaves of WT and transgenic apple plants overexpressing *MdFRK2*. To confirm the link between *MdFRK2* and melatonin, WT and *MdFRK2*-OE lines (L1, L4, and L9) were treated with 0 μM of melatonin (control) or 1,000 μM of melatonin for 14 days. Samples were obtained from fully mature leaves (the fourth to seventh leaves from the base of the stem) after 14 days of melatonin treatment. Different letters above the column indicate significant difference at *P* < 0.05 by Duncan’s test.

### *MdFRK2* Overexpression Counteracts the Hexose Accumulation Caused by Melatonin

**FIGURE 7:**
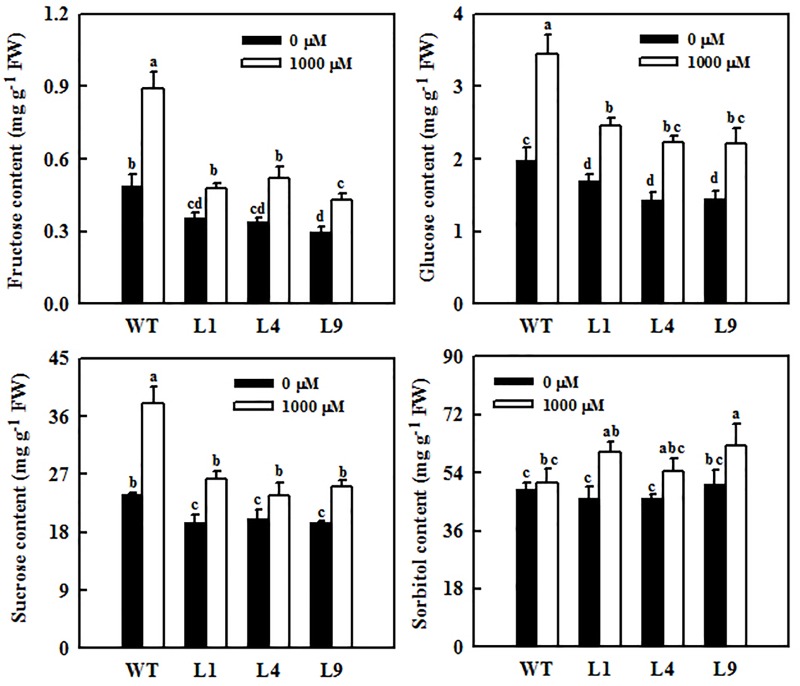
Changes in sugar concentrations in mature leaves of WT and transgenic apple plants overexpressing *MdFRK2*. Both WT and the transgenic plants were treated with 0 μM of melatonin (control) or 1,000 μM of melatonin for 14 days. Values are means of four replicates ± SD. Different letters above the column indicate significant difference at *P* < 0.05 by Duncan’s test.

*MdFRK2* was expected to be involved in melatonin-mediated sugar metabolism in apple plants. To further determine the role of *MdFRK2* in regulating sugar concentrations under melatonin treatment, we overexpressed *MdFRK2* in apple plants with the 35S promoter. Compared with those in untransformed WT lines, *MdFRK2* mRNA levels and FRK enzyme activity in the leaves of three transgenic apple lines, lines L1, L4, and L9, significantly increased (Supplementary Figure S1, Yang et al., 2018). 14 days after applying 1,000 μM of exogenous melatonin, we measured the *MdFRK2* expression and sugar concentrations of these transgenic and WT apple plants (Figure 6, 7). As expected, compared with those in WT plants treated with control solutions, the transcript levels of *MdFRK2* in WT plants treated with 1,000 μM of exogenous melatonin were reduced to 51%. However, compared with those in the three transgenic lines treated with water, *MdFRK2* expression treated with 1,000 μM of exogenous melatonin decreased by 8% for L1, 19% for L4, and 14% for L9. Similarly, enzyme analysis exhibited evident decrease in the FRK activity in melatonin-treated WT plants as compared to the WT controls (Figure 6). Exogenous melatonin application resulted in significantly high concentrations of fructose, sucrose, and glucose in the WT lines (Figure 7). However, in the mature leaves of the *MdFRK2*-overexpression (OE) lines (L1, L4, and L9), exogenous melatonin treatment led to a slight increase in fructose, glucose, and sucrose concentrations (Figure 7).

### *MdFRK2* Overexpression Counteracts the Growth-Inhibitory Effect of Melatonin

**FIGURE 8:**
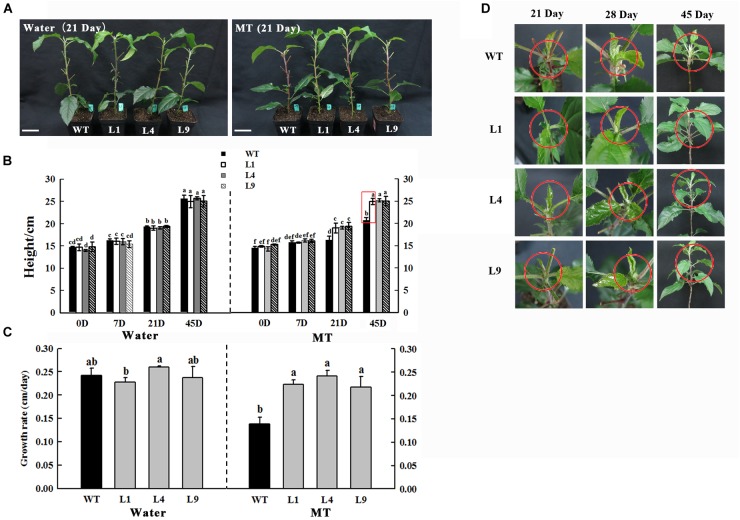
Phenotypes of WT and *MdFRK2*-OE (L1, L4, and L9) apple lines under 1,000 μM melatonin treatment. **(A)** Growth performance after 21 days of control (left) or melatonin treatment (right). **(B)** Stem height at 0, 7, 21, 45 days after melatonin treatment. **(C)** Growth rate and **(D)** shoot tip phenotypes at 21, 28, 45 days after melatonin treatment. We recorded the day on which the melatonin treatment started as the first day. The plant height was considered the average of six plants per treatment. The red circles indicate the shoot tips. Values are means of four replicates ± SD. Different letters above the column indicate significant difference at *P* < 0.05 by Duncan’s test. Scale bars represent 5 cm.

There were no obvious alterations in growth performance between WT and *MdFRK2*-OE (L1, L4, and L9) lines (Yang et al., 2018). In the presence of exogenous melatonin, the transgenic lines (L1, L4, and L9) did not show marked growth inhibition nor did their shoot tips became red after 45 days of 1,000 μM of exogenous melatonin treatment (Figure 8B–D). However, the treated WT plants appeared significantly stunted at approximately 21 days after melatonin treatment (Figure 8A).

## Discussion

### *MdFRK2* Is Involved in Melatonin-Induced Sugar Accumulation in Mature Leaves

Melatonin, acting as a plant growth and development regulator as well as a biostimulant, has shown great potential in plant physiology (Arnao and Hernándezruiz, 2015). For example, melatonin can counteract abiotic stress (Hardeland et al., 2012; Park et al., 2013; Liang et al., 2015; Wang et al., 2015). Exogenous melatonin-induced sugar accumulations (including sucrose, glucose, and fructose) have been reported in different plant species (Sarropoulou et al., 2012; Wang et al., 2015; Zhao et al., 2015a,b). Additionally, melatonin applications have also been shown to inhibit *FRK2* expression in maize plants (Zhao et al., 2015a). In accordance with the results of previous studies, we observed that, compared with applications of control solutions, applications of exogenous melatonin inhibited the expression of *MdFRK2* (Figure 3) and caused higher concentrations of sucrose, fructose, and glucose in cells (Figure 2).

Exogenous melatonin inhibited the expression of *MdFRK2*, which was confirmed by our experiments. First, *MdFRK2* transcription was inhibited by different concentrations of melatonin: the higher the melatonin concentration was, the lower the expression of *MdFRK2* and the activity of FRK (Figure 3). Second, in the presence of exogenous melatonin, GUS activity in transgenic tobacco that contained the *MdFRK2* promoter-driven reporter gene decreased as the melatonin concentration increased (Figure 5). Third, compared with those in the *FRK2*-OE lines (L1, L4, and L9), which overexpressed the *MdFRK2* gene under the control of the 35S promoter, *MdFRK2* expression and FRK activity in the WT lines was clearly inhibited in response to 1,000 μM of melatonin (Figure 6). Melatonin consistently increased fructose accumulations more in the WT lines than in the *FRK2*-OE lines (L1, L4, and L9) (Figure 7). However, expression of *MdFRK2* was also inhibited in the OE lines, suggesting that melatonin inhibit the own *MdFRK2* promoter. There should exist a yet to be determined transcription factor that is responsible for melatonin-mediated *MdFRK2* expression. Although one melatonin receptor has been identified in *Arabidopsis thaliana* (Wei et al., 2018), its signaling pathway is unknown in plant cells. The identification of the involved transcription factor will be helpful to understand the mechanism of melatonin in regulating sugar metabolism.

Fructokinase is crucial for fructose metabolism and negatively regulates the accumulation of fructose in plants (Pego and Smeekens, 2000). Expression patterns and functional analysis of FRK isoforms in tomato, rice, maize, and *Populus* suggest that FRK2 orthologs are the major FRK-encoding genes and the dominant fructose-phosphorylating enzymes in sink tissues (Kanayama et al., 1997, 1998; Pego and Smeekens, 2000; Jiang et al., 2003). *MdFRK2*, the gene that codes for the main fructose-phosphorylating enzyme in apple plants (Li et al., 2018), controls FRK activity and fructose concentrations in mature apple leaves (Yang et al., 2018). In this study, *MdFRK2* expression was inhibited by exogenous melatonin applications at both low and high concentrations (Figure 3, 5), leading to a decrease in FRK activity and an increase in fructose levels (Figure 2, 3). These results are in agreement with those of a previous report with respect to decreases in *FRK2* expression (Zhao et al., 2015a) and increases in fructose levels upon short-term exposure to a range of melatonin concentrations (10–100 μM) in maize seedlings (Zhao et al., 2015a). Taken together, these observations indicate a general role for melatonin in limiting *FRK2* expression and increasing fructose levels.

Sucrose metabolism, including its synthesis and degradation, is induced by exogenous melatonin at the gene expression and enzyme activity levels. These phenomena are reflected by the increased activities of SPS and invertase, especially under high concentrations of melatonin (100 μM) (Zhao et al., 2015a). Our results further confirm previous findings, as the SPS and NINV activity significantly increased (Figure 4). Additionally, high concentrations of melatonin were found to counteract sucrose phloem loading, as indicated by the reduced expression of the *tie-dyed2* (*Tdy2*) and *Sxd1* genes (Zhao et al., 2015a). As the closest homolog of *MdFRK2*, *LeFRK2* has been demonstrated to be required for phloem development and sugar transport (German et al., 2003). In tomato, suppression of *LeFRK2* reduced the length and width of sieve elements and resulted in their low levels of callose deposition, which restricted the downward movement of sucrose and greatly elevated soluble sugar levels in tomato source leaves (German et al., 2003). Therefore, it is possible that exogenous melatonin inhibited the expression of *MdFRK2* and then caused phloem interruption, thus leading to an abundance of sucrose in the leaves (Figure 2). The significantly increased sucrose levels may stimulate sweet immunity responses through sucrose sensing and signaling (Bolouri Moghaddam and Van den Ende, 2013).

Sugars fulfill many essential functions in plant cells (Pego and Smeekens, 2000). Plants have a system that controls sugar homeostasis to help avoid the negative effects associated with fluctuations in sugar concentrations. Excess fructose in the cytosol is harmful to plant growth and development. On the one hand, high concentrations of fructose can lead to stress reactions such as senescence. On the other hand, the inhibition of fructose phosphorylation can limit carbon flux to glycolysis and other pathways via F6P. Under high concentrations of fructose, apple plants have at least two alternative approaches to limit the production of fructose in cells: one involves sorbitol conversion to fructose via SDH catalysis (Yamada et al., 1998), and the other involves sucrose cleavage by SUSY or invertase (Pego and Smeekens, 2000; Ruan, 2014). Our results indicated that the decreased fructose concentrations in the leaves after low melatonin applications were partly limited by decreased sorbitol dehydrogenization, reflected by decreased SDH expression and activity (Figure 4). Correspondingly, the NINV activity increased (Figure 4), causing an increase in glucose levels. Interestingly, we observed opposite changes of SDH, SUSY, and NINV activities after melatonin treatment (Figure 4) compared to those in MdFRK2 overexpressing lines (Yang et al., 2018). These results indicated that carbohydrate metabolism changes in response to melatonin were mediated by MdFRK2.

### *MdFRK2* Is Involved in Growth Inhibition Mediated by Exogenous Melatonin

Compared to the control solutions and low-concentration melatonin treatments, shoot growth in response to high-concentration melatonin applications was significantly inhibited (Figure 1, 8). The observed growth inhibition effect was similar to that in previous reports on growth inhibition of maize seedlings in response to high concentrations of exogenous melatonin treatments (Zhao et al., 2015a). However, in the present study, it is interesting that the negative effect of growth was significantly alleviated in the *MdFRK2*-OE lines in response to the 1,000 μM melatonin treatment, as reflected by the similar shoot height between the *MdFRK2*-OE and WT lines (Figure 8A). Moreover, expression of *MdFRK2* and activity of FRK were suppressed slightly less in the treated *MdFRK2*-OE plants than in WT plants after 14 days of melatonin treatment (Figure 6). These phenomena indicate that *MdFRK2*-OE counteracts the effects of the high concentration melatonin-mediated growth inhibition of *MdFRK2*-OE apple plants, suggesting that the reduced expression of *MdFRK2* is an important cause of the stunted phenotype of WT plants (Figure 8).

*FRK2* plays important physiological roles in regulating plant growth and development. For example, RNAi-*MdFRK2* tomato plants displayed inhibited stem and root growth and reduced numbers of seeds per fruit (Odanaka et al., 2002). The young leaves of FRK2-antisense tomato plants showed inhibited growth and wilting because of the suppression of a particular area of active xylem (German et al., 2003). Antisense suppression of the *FRK2* ortholog in potato (*StFRK1*) inhibited aerial growth and resulted in decreased tuber yields, possibly due to reduced phloem transport (Cho and Yoo, 2011). Reduced FRK2 activity led to reduced length and width of sieve elements, thus leading to inhibited stem water conductivity and restricted downward movement of sucrose (Damari-Weissler et al., 2009). As *MdFRK2* expression is extremely high in actively growing sink tissues such as shoot tips in apple plants (Li et al., 2012), modulated expression of *MdFRK2* in shoot tips might negatively affect stem development, potentially causing growth inhibition after melatonin treatment.

Previous studies showed that hexokinase1 (*AtHXK1*) overexpression inhibits growth (Jang et al., 1997; Dai et al., 1999), and the stunted growth occurs only when *AtHXK1* expressed in photosynthetic tissues (Dai et al., 1999). Therefore, the expression of *MdHXK1* was monitored in our study. We found that the *MdHXK1* transcripts were upregulated in mature leaves, especially under high concentration of melatonin application (Figure 4). Although our results cannot provide the exact mechanism by which this occurs, these data allow us to speculate that the increased *HXK1* expression may be also involved in the inhibitory effect (Figure 1B).

It has been demonstrated that suppression of the *FRK2* orthologs resulted in increased sugar concentrations (German et al., 2003; Davies et al., 2005; Roach et al., 2012). Our data showed *MdFRK2* represents a link in the cross-talk between increased sugar concentrations and the melatonin application. Current results also support that exogenous melatonin may mimic DAMPs, counteracting both abiotic and biotic stresses through elevated leaf sweetening (Versluys et al., 2017). Spraying exogenous melatonin on leafy vegetables may be very promising in terms of agronomical applications. Especially melatonin/fructan mixtures hold great promise, as natural and sustainable alternatives for toxic agrochemicals, to be used on leafy vegetables (Versluys et al., 2017). Further investigations of the role of melatonin in leafy vegetables will be done to support or refute these theories.

## Author Contributions

JY and CZ performed the majority of experiments, analyzed, and discussed data. ZW, SS, RZ, YZ, and BM performed essential experiments. ML supervised work. JY wrote draft manuscript. All authors contributed to final manuscript.

## Conflict of Interest Statement

The authors declare that the research was conducted in the absence of any commercial or financial relationships that could be construed as a potential conflict of interest.

## Footnotes

1 http://gmd.mpimp-golm.mpg.de/

## Abbreviations

CaMV: cauliflower mosaic virus
DAMPs: damage-associated molecular patterns
F6P: fructose-6-phosphate
FRK: fructokinase
GUS: β-glucuronidase
HXK: hexokinase
MS: Murashige and Skoog medium
NINV: neutral invertase
SDH: sorbitol dehydrogenase
SPS: sucrose-phosphate synthase
SUSY: sucrose synthase.
