# Supplementary figures and images for: Melatonin-Mediated Sugar Accumulation and Growth Inhibition in Apple Plants Involves Down-Regulation of Fructokinase 2 Expression and Activity
Source: Front Plant Sci. 2019 Feb 19;10:150. doi: 10.3389/fpls.2019.00150 (PMC6389791; doi:10.3389/fpls.2019.00150)

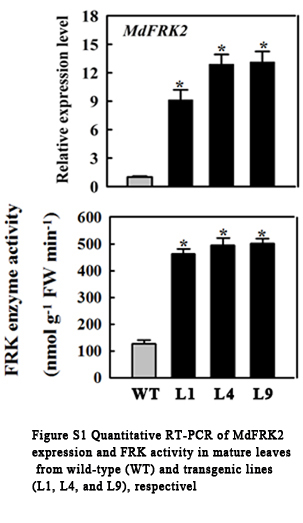

Supplement: Supplementary file 1 [file Image_1.JPEG]
