# Supplementary material for: Melatonin-Mediated Sugar Accumulation and Growth Inhibition in Apple Plants Involves Down-Regulation of Fructokinase 2 Expression and Activity
Source: Front Plant Sci. 2019 Feb 19;10:150. doi: 10.3389/fpls.2019.00150 (PMC6389791; doi:10.3389/fpls.2019.00150)
Supplement: Supplementary file 2 [file Table_1.docx]

Supplementary table 1. Primers used in this study.

| **Accession no.** | **Sequence (5’-3’)** | **usage of primers** |
| --- | --- | --- |
| MdFRK2P | F: CTGTTTAGTCATCAATTAAGATC | Vector construction for tobacco plant transformation |
|  | R: TTCACTAATGGATTATTTATGTC |  |
| qMdSDH1 | F: CTGTACCAGAGGCACCTCCGAGT | Quantitative expression of  MdSDH1 |
|  | R: TTGCATGCTCTGCTCTCTCGTTG |  |
| qMdSDH2 | F: ACA CCATCA AGA TCCTACCTT TC | Quantitative expression of  MdSDH2 |
|  | R: CATTTCATGGTC TTGAGGTAGTG |  |
| qMdCWINV1 | F: TAA CAA ATA TGT GGT GCT CCT CTG | Quantitative expression of  qMdCWINV1 |
|  | R:ACC CTA GCT GTT ATG CAC GCC T |  |
| qMdCWINV2 | F: TTC AAA GCT AAA GGC AGA CAC G | Quantitative expression of  qMdCWINV2 |
|  | R:GTA AAT CTA CAT CTA CAA AGC CAG C |  |
| qMdNINV1 | F: CTGTACCAGAGGCACCTCCGAGT | Quantitative expression of  MdNINV1 |
|  | R: TTGCATGCTCTGCTCTCTCGTTG |  |
| qMdSUSY1 | F: CTC AAG CGT GTT AAG CAA CAG | Quantitative expression of  qMdSUSY1 |
|  | R:CTG AAT GGA ACA CGA AGA ATA TC |  |
| qMdSUSY4 | F: CTGTACCAGAGGCACCTCCGAGT | Quantitative expression of  MdSUSY4 |
|  | R: TTGCATGCTCTGCTCTCTCGTTG |  |
| qMdFRK1 | F: CTGTACCAGAGGCACCTCCGAGT | Quantitative expression of  MdFRK1 |
|  | R: TTGCATGCTCTGCTCTCTCGTTG |  |
| qMdFRK2 | F: CTGTACCAGAGGCACCTCCGAGT | Quantitative expression of  MdFRK2 |
|  | R: TTGCATGCTCTGCTCTCTCGTTG |  |
| qMdFRK3 | F:AGA GTCAAGGGTATGAAGGTAGAT G | Quantitative expression of  MdFRK3 |
|  | R:CTC GTCCTGAAGCAA AGA AAGAT |  |
| qMdFRK4 | F:TCAGGATGAGGAGGGGCTACGAG | Quantitative expression of  MdFRK4 |
|  | R:CTGCTTTAAGCACTGGAGCACAGC |  |
| qMdHXK1 | F: CTG AAA GTG GTC GGG AGC AAA C | Quantitative expression of  qMdHXK1 |
|  | R:TGC ACG AGT GGC AAC TAT GTC G |  |
| qMdHXK2 | F:TGGTGGATTATACGAGCATTACA | Quantitative expression of  MdHXK2 |
|  | R:TCCAGGGTATTGTGAGTGAGA G |  |
| qMdHXK3 | F: AGA TTG TGG CGG ATG TAT GTG AC | Quantitative expression of  qMdHXK3 |
|  | R:CAA CAG TCC TCT TGC CAA AAA TG |  |
| qMdActin | F:GGACAGCGAGGACATTCAGC | Real-time PCR of actin as a reference gene in apple plants |
|  | R:CTGACCCATTCCAACCATAACA |  |
